# Mathematical Modeling Approaches for Assessing the Joint Toxicity of Chemical Mixtures Based on Luminescent Bacteria: A Systematic Review

**DOI:** 10.3389/fmicb.2020.01651

**Published:** 2020-07-31

**Authors:** Dan Wang, Shan Wang, Linming Bai, Muhammad Salman Nasir, Shanshan Li, Wei Yan

**Affiliations:** ^1^Department of Environmental Science and Engineering, Xi'an Jiaotong University, Shaanxi, China; ^2^Department of Structures and Environmental Engineering, University of Agriculture, Faisalabad, Pakistan

**Keywords:** joint toxicity, luminescent bacteria, model, QSAR, mechanism

## Abstract

Developments in industrial applications inevitably accelerate the discharge of enormous substances into the environment, whereas multi-component mixtures commonly cause joint toxicity which is distinct from the simple sum of independent effect. Thus, ecotoxicological assessment, by luminescent bioassays has recently brought increasing attention to overcome the environmental risks. Based on the above viewpoint, this review included a brief introduction to the occurrence and characteristics of toxic bioassay based on the luminescent bacteria. In order to assess the environmental risk of mixtures, a series of models for the prediction of the joint effect of multi-component mixtures have been summarized and discussed in-depth. Among them, Quantitative Structure-Activity Relationship (QSAR) method which was widely applied *in silico* has been described in detail. Furthermore, the reported potential mechanisms of joint toxicity on the luminescent bacteria were also overviewed, including the Trojan-horse type mechanism, funnel hypothesis, and fishing hypothesis. The future perspectives toward the development and application of toxicity assessment based on luminescent bacteria were proposed.

## Introduction

With the intensive industrial emissions and human activities, natural compounds and anthropogenic pollutants discharged into the environment accumulate in ecosystems, which contributed a lot to the environmental pollution. The toxicity of the pollutants, especially multi-component mixtures in a single sample are of more considerable concern due to their potential negative impact on human and other living organisms, such as modifying the stability of genetic information and inhibition of growth. The multi-component contaminants may have an adverse effect on organism even the concentration of single pollutant is less than No Observable Effect Concentration (NOEC) (Hageman et al., 2019). Furthermore, when two or more component mixtures act on a living organism simultaneously, the joint toxicity may significantly differ from the single-substance toxicity.

The sustained attention on the toxic effects of pollutants in the environment has brought forth the rapid development of sensitive and controllable monitoring techniques. In comparison with the physical and chemical analysis, that could only determine the compositions and concentration of the pollutants in the environmental samples, the biological assay could intuitively reflect the detrimental effects of contaminants toward target organisms which is of great importance and contribute to revealing the mechanisms of the toxic effects (Li et al., 2013a,b). Among various bioassay types, acute toxicity bioassays have been rapidly developing as a preferred tool for environmental risk assessment in recent decades which can detect the adverse effects of a single component or multi-component mixtures in a short period and determine the relationship between dose and effect of toxic substances. Acute toxicity bioassays generally can be conducted with both prokaryote and eukaryote, containing bacteria (Ma et al., 2018; Rao et al., 2019; Feng et al., 2020), algae (Costa et al., 2018; Staveley et al., 2018), invertebrates (Chen et al., 2020; Sackey et al., 2020; Vilela et al., 2020), vertebrates (Clemow and Wilkie, 2015; Thornton et al., 2017; Barron et al., 2018; Ivey et al., 2019), and so on. Inevitably, the application of multicellular organisms exhibits the undesirable limitations of relatively longer operation time and higher cost. Therefore, microbial bioassays, especially with luminescent bacteria increased widely interests in the acute toxicity bioassays due to its operational simplicity, less time and cost consuming, higher sensitivity, specificity, and reproducibility (Conforti et al., 2008; Skotti et al., 2014; Yang et al., 2016).

With the growing number of disparate predictions available to perform multi-component mixtures biotoxicity analyses with luminescent bacteria, the choice of methods presents a major challenge to researchers as there is no review of comparative methods currently available. In this review, we summarize the occurrence of bioluminescent assays and the prevalence of determination of joint toxicity of multi-component mixtures and describes the various types of models involved in joint toxicity prediction. The representative methods for the prediction and evaluation of acute joint toxicity are overviewed including graphical method, joint effect indexes, and integrative models. Furthermore, the QSAR method as a hotspot in joint toxicity researches, are also presented in detail.

## The Physiological Mechanism and Development of Toxic Bioassay by Luminescent Bacteria

Currently, luminescent bacteria have been applied worldwide in the bioassays of toxicity assessment, including *Aliivibrio fisheri* (*A. fischeri*, previously identified as *Vibrio fisheri* or *Photobacterium fisheri*), *Photobacterium phosphoreum, Vibrio qinghaiensis, Vibrio harveyi* and so on (Urbanczyk et al., 2007). Luminescent bacteria that could emit visible bioluminescence under certain physiological conditions were primarily isolated from the ocean environment except for *V. cholerea* and *V. qinghaiensis* sp. Nov (Zhu et al., 1994; Ma et al., 2014).

## Physiological Mechanism of Bioluminescence From Luminescent Bacteria

**Figure 1:**
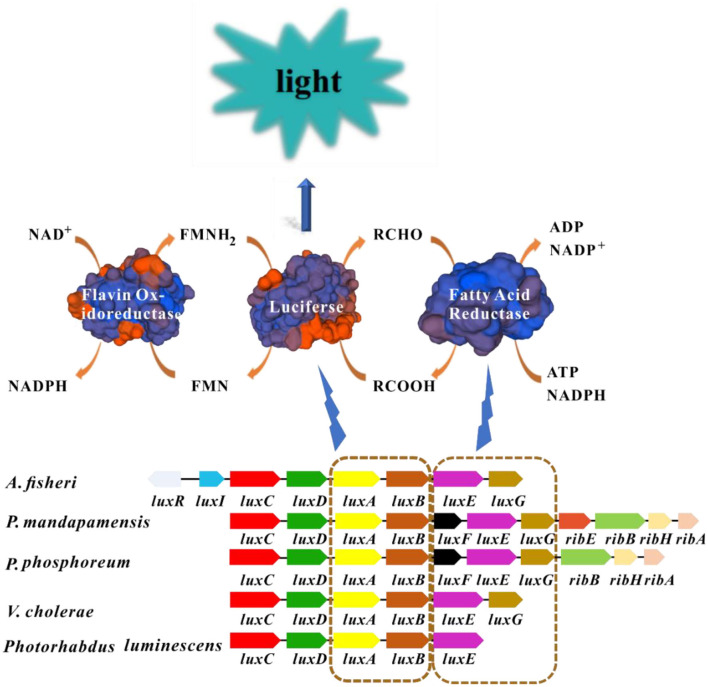
Luminescence mechanism of luminescent bacteria. Blocks with the same color in different bacteria represent homologous genes. The *luxA* and *luxB* encode luciferase, *luxC, luxD*, and *luxE* encode the fatty acid reductase complex. The structures of enzymes in this picture are modeled by SWISS-MODEL (https://www.swissmodel.expasy.org/). FMNH_2_ and long-chain fatty aldehyde are oxidized by luciferase enzyme into FMN and long chain fatty acid, respectively. This process is accompanied by the production of blue-green light.

The bioluminescence mechanism of luminescent bacteria has been fully studied and characterized (Inouye, 1994; Moore and James, 1995; Bourgois et al., 2001; Vetrova et al., 2007; Nijvipakul et al., 2010; Bergner et al., 2015; Brodl et al., 2018; Tanet et al., 2019). Generally, luciferase, reduced flavin mononucleotide (FMNH_2_), oxygen (O_2_), and long-chain fatty aldehyde (RCHO) are prerequisite for the luminescence emitted by luminescent bacteria in which flavin mononucleotide (FMN) was reduced to FMNH_2_ by the catalyzation of NAD(P)H: FMN oxidoreductase. Then FMNH_2_ and molecular oxygen will bind with luciferase which has flavin reductase activity to form the intermediate_._ Subsequently, the intermediate will decompose in the presence of aldehyde, FMNH_2_ are oxidized into FMN and H_2_O by the catalyzation of luciferase enzyme, along with the emitting of continuous blue-green light (490 nm) (Figure 1) (Inouye, 1994).

The enzyme involved in bioluminescence in luminescent bacteria is commonly encoded by *lux* genes which are usually clustered together as *lux* operon (*luxCDABE*) (Matheson and Lee, 1981). Luciferase possesses α and β subunits, that are encoded by *luxA* and *luxB* genes, respectively. Moreover, the α subunit which exhibits major catalytic activity is similar to the β subunit in amino acid sequence (around 30%) (Sharifian et al., 2017). In general, the organization of *lux* genes and the amino acid sequences from different luminescent bacteria are similar which indicates their origin from common ancestor and conservation in evolution. Fatty acid reductase complex is encoded by *luxC, luxD*, and *luxE*, which is responsible for the transformation between long-chain fatty acids and fatty aldehydes (Figure 1). In certain luminescent bacteria such as *Aliivibrio* species, the presence of regulatory genes *luxI* and *luxR* are detected which link bioluminescence with quorum sensing (QS) (Miyashiro and Ruby, 2012). The gene *luxI* encodes the enzyme required for the synthesis of the autoinducer molecule, and LuxR is the autoinducer-dependent transcription factor, the binding of autoinducer-LuxR to the promoter will initiate the transcription of *lux* operon (Engebrecht and Silverman, 1984; Fuqua et al., 1994). Some additional *lux* genes including *luxG, luxF* and *luxH* have also been found in some luminescent *lux* operons, but there is no evidence to confirm that these genes were directly related to bioluminescence (O'Grady and Wimpee, 2008; Bergner et al., 2015; Deeva et al., 2019).

## Development Application of Toxicity Assessment by Luminescent Bacteria

The toxicity bioassay with luminescent bacteria has been successfully employed in the comprehensive ecotoxicity assessments. In 1978, Beckman Company of the United States first commercialized the toxic bioassay as “Microtox®” with *A. fischeri*, the sensitivity of which was comparable to the 96 h acute toxicity test of fish (Flokstra et al., 2008). Since then, the toxicity assessment by luminescent bacteria has been developing rapidly toward higher sensitivity, accuracy, reproducibility, anti-interference, and extensive application. As a comprehensive indicator, luminescent bacteria have been successfully applied for monitoring the ecotoxicity of the natural environment, including wastewater, river water, sewage sludge, landfill leachate, and so on (Jarque et al., 2016; Pino-Otín et al., 2019). Zhang et al., examined the toxicity of surface water from the Huangpu River of China by *V. qinghaiensis* sp.-Q67, and found the seasonal variation of toxicity (Zhang et al., 2015). Likewise, Jarque et al. held the view that *V. fishieri* luminescence assay to evaluate the toxicity associated with sediments from the Morava River and its tributary Drevnice River followed seasonal patterns (Jarque et al., 2016). Luminescent bacteria toxicity bioassay could also be carried out to evaluate the ecological risk of wastewater before and after diverse treatment processes, including ozonation, Fenton, UV, electrochemical degradation, biological process, and so on (De Schepper et al., 2012; Hamdi El Najjar et al., 2014; Lütke Eversloh et al., 2014; Wei et al., 2015; Nebout et al., 2016; Xu et al., 2018).

No confined to aqueous bodies, luminescent bacteria bioassay are also conducted in another environmental medium. Aammi et al. evaluated the acute toxicity and genotoxicity levels of atmospheric particulate matter (PM) samples from three districts in Istanbul, Turkey. *V.fischeri* was used to test the biotoxicity of the collected ambient air samples. The study identified significant locational and seasonal differences in the toxicity level of PM_2.5−10_ extracts. In terms of seasonal differences, more toxic and anthropogenic particle samples present in winter. Most of the toxic PM samples were identified in central urban, it can be attributed mainly to the inner-city and transit traffic emissions (Aammi et al., 2017). Moreover, Microtox® system along with *V.fischeri* has also been applied to determine the ecotoxicity of PM emitted from three different light-duty vehicles, the results demonstrated that PM from both gasoline and diesel vehicle equipped with the silicon-carbide catalyzed diesel particle filter (CDPF) appeared higher toxicity on a per mass basis than the corresponding vehicle with conventional exhaust, which indicated the decrease of PM emission level was not equated to the reduction of toxicity (Vouitsis et al., 2009). To reveal the influence factors on the formation of environmental persistent free radicals (EPFRs) in soil and the associated biotoxicity, Zhang et al. mimicked the formation of the EPFRs in the catechol-contaminated soil, they concluded that significantly inhibited the bioluminescence of *P. phosphoreum* when the concentration of EPFRs reached the maximum at pyrolysis temperature of 300. It could be speculated that the production of ·OH or reactive oxygen species (ROS) may be the dominant contributor to its acute toxicity to luminescent bacteria (Zhang et al., 2019b).

**Figure 2:**
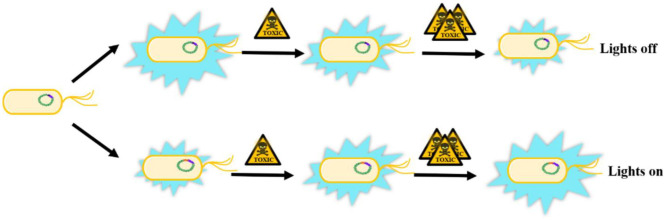
The biosensor patterns in toxicity assessment based on recombinant luminescent bacteria. Lights off: the luminescence will be emitted continuously and decrease with the increasing quantity of toxic substances. Lights on: *lux* genes are fused with inducible promoters which could be transcribed and expressed under the expose of specific chemicals and the luminescence will increase with the increasing quantity of specific toxic chemicals. Chromosome, *lux* genes and promoters are presented with green, purple and orange color respectively.

The conventional biotoxicity evaluation methods based on luminescent bacteria suffered from only being able to detect the overall toxicity without selectivity. A great deal of previous research has focused on specific toxins toxicity evaluation of target substances to overcome the drawback. Surveys such as that conducted by Huang et al. (2019a) have shown that dual detection by a combination of luminescent bacteria and aggregation aggregation-induced emission (AIE) probe could detect bioaccumulated Hg^2+^. The toxic effect of Hg^2+^ on *P. phosphoreum* give rise to quenching its bioluminescence inside the bacteria. Moreover, the AIE-active probe 2-AFN-I entered the damaged bacteria and selectively bound to Hg^2+^, which would turn-on the fluorescence and complete the overall dual detection (Huang et al., 2019a). What's more, microbial biosensors as one of the most significant applications of luminescent bacteria has been developing gradually with the incomparable advantages of selective identification and quantification of specific substances directly in air and water. Microbial biosensors are usually composed of immobilized microorganisms (recognition element) in the transducer to produce a measurable signal such as current, potential, or optical a signal, which can be further amplified and analyzed. Since 1997, Boyd and Mowat confirmed the change of luminescence emitted by living microorganisms in the presence of a target analyte (Boyd et al., 1997), bioluminescence genes (*lux*) have been recognized as an effective and preferable reporter system of biosensors for toxicity evaluation. The *luxCDABE* genes have been transferred into specific bioreporter bacteria under the control of a particular promoter to integrate into the recognition element of a biosensor. These recombinant luminescent bacteria can be divided into two categories according to expression pattern (Figure 2). If the *lux* genes are transferred into the constitutive expression system, the luminescence will be emitted continuously and decrease with the increasing quantity of toxic substances (“lights off”), which is non-specific and suitable for the total toxicity assessment of the mixture. Most natural bioluminescent bacteria belong to this group (Belkin, 2003; Gu et al., 2004). In the other group, *lux* genes are fused with inducible promoters which can be activated under the exposure of specific chemicals. Therefore, the produced luminescence will increase with the increasing amount of specific toxic chemicals (“lights on”). Most of the microbial biosensors based on luminescent bacteria could be classified as this specific group (Belkin, 2003). Nowadays, biosensors are rapidly developing toward higher sensitivity, specificity, reproducibility, automation and high throughput (Conforti et al., 2008; Skotti et al., 2014; Yang et al., 2016). Furthermore, some specific biosensors with recombinant bioluminescent bacteria have also been designed to detect cell damage, like DNA damage, protein damage, cell membrane damage and stress response (Ahn et al., 2009; Sazykin et al., 2016; Jiang et al., 2017).

## Evaluation and Prediction of Joint Toxicity of Mixed Substances

The multi-component mixtures could cause combined toxicity which is distinct from independent effect, due to the apparent differences between the toxic mode of actions (TMOA) and the interactions among constituents. Even if the characteristics of each component are acquainted, the integrative effect of the mixture could not be directly inferred. Therefore, the evaluation and prediction of the joint effect of mixed contaminants have been attracting increasing interests. In the research field of environmental toxicology, the most reported studies on joint toxicity focused on the evaluation of the combined effects of components in the mixed system (Monosson, 2005). According to the different interaction modes of the constituents in a mixture, combined toxicity can be categorized into diverse effects, including antagonism (ANT), additive (ADD), independent (IND), and synergistic (SYN) effects (Plackett and Hewlett, 1952). With the development of toxicology and mathematics, the existing qualitative evaluation methods neither meet the requirements of determining the toxicity contribution of a single component to mixed pollutants nor meet the requirements of predicting the toxicity of a mixture of specific known components. Therefore, more efforts have been made on the quantitative assessments and predictions of joint toxicity of multi-component mixture (Altenburger et al., 2003). Generally, the combined toxicity of the multi-component was determined using a fixed ratio design. Always, the EC_50_ value of each component within the mixture is constituted a fixed ratio design to evaluate the joint effect. The EC_50_ value is defined as the concentration that produces a 50% decrease in light emission, obtained from the concentration-response relationship, which could calculate from regression models (Table 1). Next, the joint toxicity of multi-component mixtures would be predicted using disparate models when mastering some knowledge of EC_50_ of an individual component.

**Table 1:** Classical regression models for calculating the concentration-response relationships.

**Name**	**Function regression expression**
Probit	$P\left(Con\right)=\frac{1}{2\pi }\overset{{\;}^{\theta_{1}+\theta_{2}\mathrm{lg}\left(Con\right)}}{\underset{-\infty }{\int }}\exp\left(-\mu^{2}/2\right)d\mu =\Phi \left(\theta_{1}+\theta_{2}\mathrm{lg}\left(Con\right)\right)$
Logit	$P\left(Con\right)\text{=1}-\frac{1}{1+{\left(\theta_{1}Con\right)}^{\theta_{2}}}$
Morgan-mericier	$P\left(Con\right)=\frac{1}{\left(1+\text{exp}\left(-\theta_{1}+\theta_{2}\mathrm{lg}\left(Con\right)\right)\right)}$
Weibull/Gomertz	*P*(*Con*) = 1−exp(−exp(θ_1_ + θ_2_lg(*Con*)))
Generalized logit 1	$P\left(Con\right)=\frac{1}{{\left(1+\exp\left(-\theta_{1}-\theta_{2}\mathrm{lg}\left(Con\right)\right)\right)}^{\theta_{1}}}$
Generalisied logit 2	$P\left(Con\right)=1-\frac{1}{{\left(1+\exp\left(\theta_{1}+\theta_{2}\mathrm{lg}\left(Con\right)\right)\right)}^{\theta_{3}}}$
Aranda-ordaz	$P\left(Con\right)=1-\frac{1}{{\left(1+\exp\left(\theta_{1}+\theta_{2}\mathrm{lg}\left(Con\right)\right)/\theta_{3}\right)}^{\theta_{1}}}$
Logit with box-cox-transformation	$P\left(Con\right)={\left(1+\exp\left(-\theta_{1}-\theta_{2}\frac{Con^{\theta_{3}}-1}{\theta_{3}}\right)\right)}^{-1}$
Weibull with box-cox- transformation	$P\left(Con\right)=1-\exp\left(-\exp\left(\theta_{1}+\theta_{2}\frac{Con^{\theta_{3}}-1}{\theta_{3}}\right)\right)$
Probit with box-cox- transformation	$P\left(Con\right)=\Phi \left(\theta_{1}+\theta_{2}\frac{Con^{\theta_{3}}-1}{\theta_{3}})\right)$

*Con denotes the concentration of the tested substance, P (Con) is the mean effect of the model and θ_1_, θ_2_, θ_3_ are estimated model parameters. Where Φ (y) is the cumulative normal (Gauss) distribution, meaning that the probability that a standard normal random variable is less than y*.

## Graphical Methods

### Isobologram

**Figure 3:**
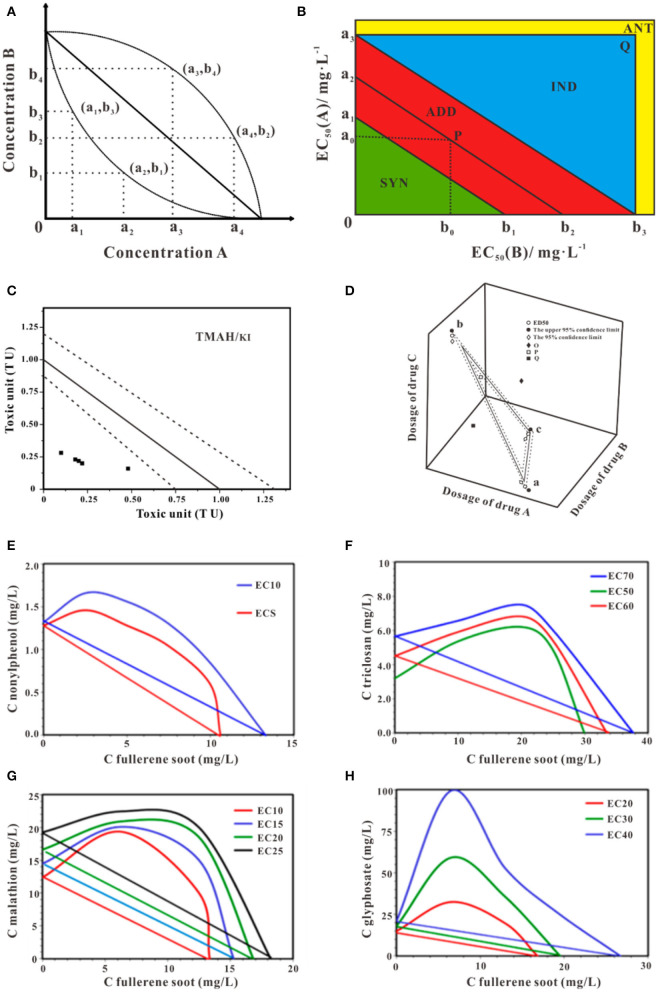
Schematic isobologram for toxicity assessment. **(A)** The effect of components A+B at random mixture ratio in the Cartesian plane at a given effect level should be theoretically located on the straight line. The upward and downward concave lines act antagonistically and synergistically, respectively. **(B)** Improvement equivalent effect picture of joint toxicity. With different toxic ratios, the isobologram method may yield distinct joint effects (Ding et al., 2015). **(C)** Isobologram of TMAH and KI mixture. The solid straight line indicates theoretical additive toxicity. Dot lines indicate 95% confidential interval of the theoretical additive effect (Mori et al., 2015). **(D)** The theoretical isobologram of the combination of three substances (Huang et al., 2019b). **(E)** Isobologram for a mixture of fullerene soot and nonylphenol (Sanchis et al., 2016). **(F)** Isobologram for a mixture of fullerene soot and triclosan (Sanchis et al., 2016). **(G)** Isobologram for the mixture of fullerene soot and malathion (Sanchis et al., 2016). **(H)** Isobologram for the mixture of fullerene soot and glyphosate (Sanchis et al., 2016). Adapted and modified with permission from Ding et al. (2015), Mori et al. (2015), Huang et al. (2019b) and Sanchis et al. (2016).

Isobologram was originally developed by Loewe and Muischnek (1926). This method can be used to determine whether the multi-component mixtures behaved in similar modes of action. The straight-line presents the theoretical additive effect of the two components connect two individual effects caused by the fixed concentration. The accuracy of the estimated intercepts of the theoretical isobole with the axis affects the degree of deviation of observed data and experimental data. In many cases, the deviation may result in inadequacy predication. Bernbaumhas put forward interaction index (CI) to directly present the effects on non-interactive multi-compounds mixtures from the dose-effect curve of the individual compound (Berenbaum, 1981). The graphical representation is shown in Figure 3A. In the binary mixture, the equation of the CI is showed in Equation (1). In this equation, a_1_ and b_1_ are the dose of components in the mixture, A and B are the dosed of the individual agents giving rise to the same effects as the mixtures. The combination index (CI) determinates the joint effect of the combinations, that shows in **Figure 6**.

(1) $$\begin{array}{l} \text{CI}=\frac{a_{1}}{A}+\frac{b_{1}}{B} \end{array}$$

Applied with the 95% confidence interval to simplify the process of the judgment, Ding proposed an improvement approach to characterize the binary toxicities of mixtures and applied this judgment to evaluate the joint toxicity of antibiotics successfully (Figure 3B) (Ding et al., 2015). The EC_50_ of compounds A and B are presented by a_2_ and b_2_, respectively. (a_1_, a_3_) is the 95% confidence limit of a_2_, while (b_1_, b_3_) is the 95% confidence limit of b_2_. The values a_0_ and b_0_ represent the equivalent concentration of EC_50_ for mixture A and B. According to the location of P (a_0_, b_0_), the joint toxicity effect of mixture A and B can be judged. If P is located between line a_1_b_1_ and a_3_b_3_, the joint effect is additive, if P is underline a_1_b_1_, the joint toxicity is synergy if P is above line a_3_b_3_ and in the triangle a_3_b_3_Q, the joint toxicity is independent and if P is located outside the rectangle a_3_Ob_3_Q, the joint effect is antagonistic. Much of the available literature on isobologram deals with the question of mixtures toxicity of multi-component. Using the experimental data of poly and perfluoroalkyl substances (PFAS) based on an amphibian fibroblast cell line, Hoover et al. employed the isobolograms to ascertain the interaction type of the binary mixtures of PFAS, the additive effects were shown in most combination tests (Hoover et al., 2019). Mori et al. in his research conducted isobologram analysis which based on toxic unite (TU) to explore the synergism of the mixture of Tetramethylammonium hydroxide (TMAH) and potassium iodide (KI) to *Daphnia magna*, using four mixture ratios of TMAH and KI (4:1, 3:2, 1:1, 1:4), as shown in Figure 3C (Mori et al., 2015). Similarly, the bioluminescent inhibition of *V. fisheri* by exposure to binary mixtures of fullerene soot and nonylphenol (Figure 3E), triclosan (Figure 3F), malathion (Figure 3G), and glyphosate (Figure 3H) using isobologram were discussed, indicating antagonism (Sanchis et al., 2016). Moreover, if three substances are used together, the equivalent surface is shown in Figure 3D. The method of judging the toxicity effect of the three substances is the same as that of a binary substance (Huang et al., 2019b). One should be cautious, isobologram is a graphical method so that variability of date can-not be reflected in this approach and visualization of the effect of mixtures is different, and the graphics draw require large data sets to produce sufficiently reliable results (Berenbaum, 1981; Cassee et al., 1998; Meadows et al., 2002). It is worth mentioning isobologram is unconcerned about chemical structures or modes of action of the individual chemical.

### Response Surface

Response or effect surface methodology is based on mathematical relationships, which is obtained by multiple linear regression. The zero interaction responses surface for dose additivity method is depicted in the Equation (2).

(2) $$\begin{array}{l} \frac{d_{A}}{\alpha_{A}{\left(\frac{{E^{0}}_{AB}}{1-{E^{0}}_{AB}}\right)}^{\frac{1}{\mu_{A}}}}\text{+}\frac{d_{B}}{\alpha_{B}{\left(\frac{{E^{0}}_{AB}}{1-{E^{0}}_{AB}}\right)}^{\frac{1}{\mu_{B}}}}=1 \end{array}$$

where *d*_A_, *d*_B_ represents the dose of components A and B in the mixture. *E*${\;}_{AB}^{0}$ is the zero interaction combinations effect. The variable α and μ mean the EC_50_ and slope, respectively.

**Figure 4:**
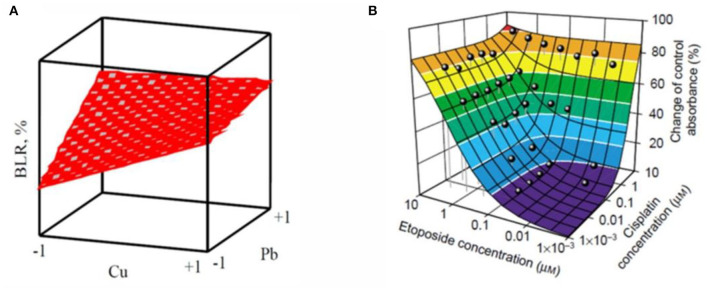
Two examples of response surface modeling. **(A)** is the response surface for Cu^2+^- Pb^2+^ mixture with a metal concentration in coded units (Ren et al., 2004). **(B)** is a color-coded (Loewe) additivity surface which compared with experimental combination data for the cytotoxic effect of etoposide–cisplatin combinations on NCI-H226 cell lines in ACL-4 medium (Groten et al., 2001). Adapted and modified with permission from Ren et al. (2004) and Groten et al. (2001).

In the case of significant, the response surface for binary components mixtures can be displayed in three dimensions. The concentration of the two substances is expressed as X-axis and Y-axis, respectively. The combined effect of the two substances is showed as Z-axis. The response surface is displayed by different mathematical model fitting. Neither the synergistic effect nor antagonistic effect of the response surface is flat, because of the two components non-existence of interaction. Ren et al. analyzed the binary effect of copper and lead toward luminescent bacteria based on the response surface, as shown in Figure 4A. They found the transition between different effects with the concentration of metal in the mixtures (Ren et al., 2004). Similarity, Tsai et ai. used an isobologram analysis to test combinations of two drugs etoposide and cisplatin against NCI-H226 cell lines, the results obtained from the preliminary analysis are shown that there was no synergism between etoposide and cisplatin at the 50% level, the sketch modified from Groten et al. (Figure 4B) (Tsai et al., 1989; Groten et al., 2001). It should be noted if predictions for doses extend intended range may result in deviations. Moreover, the approach is related to the relatively large number of parameters that must be determined by non-linear regression techniques, which requires a lot of experiments.

### Methods Based on Toxic Unite

Sprague and Ramsay first proposed Toxic Unit (TU) concept to describe the contribution of the individual component to the overall effect (Sprague and Ramsay, 1965). In the luminescent bacteria toxicity assay, the relative luminescence intensity value (RLU) decreased with the toxic substance mass concentration increased. While TU is derived from scaling a measured compound concentration to its inherent EC_50_ (Fulladosa et al., 2004). The TU concept has been applied to weigh the effect of single chemicals for the assessment of joint toxicity, can be calculated by Equations (3–5):

(3) $$\begin{array}{l} \text{T}{\text{U}}_{i}=\frac{c_{i}}{EC_{50,i}} \end{array}$$

(4) $$\begin{array}{l} \text{M}=\overset{\text{n}}{\underset{i=1}{\sum }}\text{T}{\text{U}}_{i}\text{=}\frac{c_{1}}{EC_{50,1}}+\frac{c_{2}}{EC_{50,2}}+...+\frac{c_{n}}{EC_{50,n}} \end{array}$$

(5) $$\begin{array}{l} {\text{M}}_{0}=\frac{\text{M}}{{\left(\text{T}{\text{U}}_{i}\right)}_{\max}} \end{array}$$

where c_*i*_ represents the concentration of component *i*. When the mixture is at its EC_50_, TU_i_ is the toxic unit of *i*, M is the sum of the toxic units, (TU_*i*_)_max_ is the maximum toxic unit of the mixture, and M_0_ is the ratio of M and (TU_*i*_)_max_.

**Figure 5:**
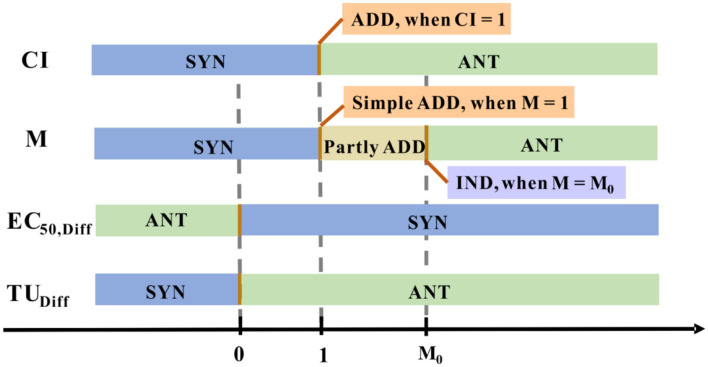
The joint effect is quantified numerically using different methods. Distinctive colored blocks represent a distinctive joint effect type.

In such a way, M quantified the joint effect that showed in Figure 5 (Chen and Huang, 1996). This approach is relatively simple and can roughly reflect the relationship between the reaction mixtures, the central premise of this concept is that each of these components alone cannot produce any effect.

Based on the concept of TU, some other joint effect indexes have been proposed to perfect the evaluation system (Table 2). The evaluation expressions are concise and the model expectations are replaced by a value to quantify the degree of deviation. However, there are still some inevitable limitations: (1) Only EC_50_ value is provided in the indexes, the evaluation about different levels are unavoidable lost. (2) Similar to the isobologram method, experimental variability also consists of these joint effect indexes method, it is unlikely to meet calculated responses precisely (Altenburger et al., 2003). In some cases, the combination index associated with the isobologram was put forward to address the issue of combination ecotoxicological assessments, effectively (Chou, 2006; Wang et al., 2015d; Godoy et al., 2019).

**Table 2:** Criteria for the judgment of joint effects of different evaluation indexes.

**Type of index and type of interaction**	**Joint effect index**		
	**Additivity index (AI)**	**Similarity parameter (λ)**	**Mixture toxicity index (MTI)**
Mathematical model	The expression depicted as Equation (4) if M <1, AI = 1/M−1, if M > 1, AI = 1/M + 1, if M = 1, AI = M−1	${\sum \left(\text{T}{\text{U}}_{i}\right)}^{\left(\text{1}/\;\lambda \;\right)}=1$	$\text{MTI}=1-\frac{\text{logM}}{\text{log}{\text{M}}_{\text{0}}}$
SYN	AI > 0	λ > 1	MTI > 1
Simple ADD	AI = 0	λ = 1	MTI = 1
IND	–	–	MTI = 0
ANT	AI <0	0 < λ <1	MTI <0
Partly ADD	–	–	0 < MTI <1
Reference	L, 1977	Prakash et al., 1996	Konemann, 1981

–* the effect is inexistence when used this type*.

Apart from the fore-mentioned joint effect indexes, other mathematical models can predict and analyze the joint effect of the binary chemical mixtures. The models always analyze the difference between the calculated value from the model and the observed value from the actual experiments. In this review, two popular evaluation approaches are recommended in detail.

The first approach (referred to statistical approach 1) was proposed by Ribo and Rogers (1990), which assumed that two in a mixture have the same TMOA do not react with each other. The interactive toxicity in that binary mixtures was studied by calculating the difference (EC_50,Diff_) between the observed toxicities (EC_50,Obs_) of the binary mixtures with the calculated toxicities (EC_50,Calc_). The computed mixture toxicities were calculated as Equations (6) and (7).

(6) $$\begin{array}{l} \text{E}{\text{C}}_{\text{50, Calc}}=\frac{{\text{C}}_{\text{M}}}{\frac{{\text{C}}_{\text{x}}}{\text{E}{\text{C}}_{\text{50,x}}}\text{+}\frac{{\text{C}}_{\text{y}}}{\text{E}{\text{C}}_{\text{50,y}}}} \end{array}$$

(7) $$\begin{array}{l} \text{E}{\text{C}}_{\text{50, Diff}}\text{= E}{\text{C}}_{\text{50, Cale}}\text{- E}{\text{C}}_{\text{50,Obs}} \end{array}$$

where C_M_ is the sum of the concentrations of the substances in the binary mixture (C_x_ and C_y_) and the EC_50,x_, and EC_50,y_ is the effective concentrations representing the observed toxicity of each substance separately. The obtained EC_50,Diff_ value implies the type of interaction between the two substances in the mixture (Figure 5). ADD effect is expressed when the difference is considered statistically non-significant (at a 95% confidence level), the definition of ADD effect is also applied to the following statistical approach 2. With this approach, Fulladosa et al. predicted the toxicity of the possible binary equitoxic mixtures of Co, Cd, Cu, Zn, and Pb to *A. fischeri*. The research showed the most combined effect of the metals was antagonistic for Co-Cd, Cd-Zn, Cd-Pb and Cu-Pb, while the synergistic effect for Co-Cu and Zn-Pb and merely additive in some cases (Fulladosa et al., 2005).

The second popular approach (referred to statistical approach 2) postulated that there may be interactions between two chemicals that act independently on biological models through different mechanisms. For that reason, the computed mixture toxicities can be expressed as new toxicity units (TU), defined as TU= 100/EC_50_, the definition is different from the above classical TU. The results can be calculated by Equations (8) and (9).

(8) $$\begin{array}{l} {\text{H}}_{\text{o}}\text{=T}{\text{U}}_{{\text{H}}_{\text{0}}}\text{(X+Y)=T}{\text{U}}_{\text{X}}\text{+T}{\text{U}}_{\text{Y}} \end{array}$$

(9) $$\begin{array}{l} \text{T}{\text{U}}_{\text{Diff}}\text{=T}{\text{U}}_{{\text{H}}_{\text{o}}}\text{-T}{\text{U}}_{\text{Obs}} \end{array}$$

where TU_X_ and TU_Y_ are the toxicity units of X and Y, TU_Ho_ (X+Y) is the toxicity units representing the a priori expected toxicity of a mixture of two components X and Y. The interaction effect was indicated by TU_Diff_ value (Figure 5). Fulladosa et al. studied binary As (V) species mixtures based on the statistical analysis of TU_Obs_ and TU_Ho_, they noted that As (V) species mixture interaction types were ANT at the pH of 6 and 7 (Fulladosa et al., 2004).

Based on the derivative models associated with TU, several studies have been conducted for ecotoxicity assay of diverse pollutants toward luminescent bacteria, which are categorically reviewed as shown in Table 3.

**Table 3:** Characterization of the acute toxicity of multi-component mixtures assessments associated with TU by luminescent bacteria.

**Categories**	**Test substances**	**Test organism**	**Mixture ratio**	**pH**	**Indicators**	**EC_**50**_ (Experimental)**	**Significance**	**Joint effect**	**References**
Heavy metals mixtures	Cd (II) + Cu (II)	*P. phosphoreum*	EC_50_: EC_50_	5.5–6	MTI/TU	8.581 mg/L	0.969	ANT/ANT	Zeb et al., 2016
	Cd (II) + Pb (II)	*P. phosphoreum*	EC_50_: EC_50_	5.5–6	MTI/TU	2.076 mg/L	0.9036	ANT/ANT	Zeb et al., 2016
	Cu (II) + Pb (II)	*P. phosphoreum*	EC_50_: EC_50_	5.5–6	MTI/TU	1.781 mg/L	0.9266	ADE^*^/ADE^*^	Zeb et al., 2016
	Cd (II) + Cu (II) + Pb (II)	*P. phosphoreum*	EC_50_: EC_50_	5.5–6	MTI/TU	2.443 mg/L	0.9548	ADE^*^/ADE^*^	Zeb et al., 2016
	Pb (II) + Cd (II)	*V. qinghaiensis*	EC_50_: EC_50_	–	TU	1.2 TU	0.9719	ANT	Xiong et al., 2007
	Pb (II) + Cr (VI)	*V. qinghaiensis*	EC_50_: EC_50_	–	TU	2.47 TU	0.93	ANT	Xiong et al., 2007
	Cd (II) + Cr (VI)	*V. qinghaiensis*	EC_50_: EC_50_	–	TU	0.67 TU	0.9554	SYN	Xiong et al., 2007
	Co (II) + Cd (II)	*A. fischer*	EC_50_: EC_50_	6	A1/A2	0.755 mmol/L	0.001/0.031	ANT/ANT	Fulladosa et al., 2005
	Co (II) + Zn (II)	*A. fischer*	EC_50_: EC_50_	6	A1/A2	0.666 mmol/L	0.098/0.069	ADD/ADD	Fulladosa et al., 2005
	Co (II) + Cu (II)	*A. fischer*	EC_50_: EC_50_	6	A1/A2	0.258 mmol/L	1.2E^−6^/6.4E^−6^	SYN/SYN	Fulladosa et al., 2005
	Co (II) + Pb (II)	*A. fischer*	EC_50_: EC_50_	6	A1/A2	0.359 mmol/L	0.284/0.12	ADD/ADD	Fulladosa et al., 2005
	Cd (II) + Zn (II)	*A. fischer*	EC_50_: EC_50_	6	A1/A2	0.126 mmol/L	0.002/0.004	ANT/ANT	Fulladosa et al., 2005
	Cd (II) + Cu (II)	*A. fischer*	EC_50_: EC_50_	6	A1/A2	0.078 mmol/L	0.181/0.113	ADD/ADD	Fulladosa et al., 2005
	Cd (II) + Pb (II)	*A. fischer*	EC_50_: EC_50_	6	A1/A2	0.152 mmol/L	0.001/2.8E^−6^	ANT/ANT	Fulladosa et al., 2005
	Zn (II) + Cu (II)	*A. fischer*	EC_50_: EC_50_	6	A1/A2	0.012 mmol/L	0.735/0.440	ADD/ADD	Fulladosa et al., 2005
	Zn (II) + Pb (II)	*A. fischer*	EC_50_: EC_50_	6	A1/A2	0.006 mmol/L	0.023/0.001	SYN/SYN	Fulladosa et al., 2005
	Cu (II) + Pb (II)	*A. fischer*	EC_50_: EC_50_	6	A1/A2	0.007 mmol/L	0.002/3.1E^−6^	ANT/ANT	Fulladosa et al., 2005
	Cu (II) + Zn (II)	*E. coli* HB101pUCD607	0.48: 1 (mg/L: mg/L)	7	TU/AI/MTI	8.54 mg/L	–	ANT/ANT/ANT	Zhou et al., 2018
	Cu (II) + Zn (II)	*E. coli* HB101pUCD607	1.44: 1 (mg/L: mg/L)	7	TU/AI/MTI	8.14 mg/L	–	ADD^*^/ANT/ADD^*^	Zhou et al., 2018
	Cu (II) + Zn (II)	*E. coli* HB101pUCD607	4.33: 1 (mg/L: mg/L)	7	TU/AI/MTI	7.89 mg/L	–	ANT/ANT/ANT	Zhou et al., 2018
	Zn (II) + Hg (II)	*E. coli* HB101pUCD607	12.73: 1 (mg/L: mg/L)	7	TU/AI/MTI	9.51 mg/L	–	ANT/ANT/ANT	Zhou et al., 2018
	Zn (II) + Hg (II)	*E. coli* HB101pUCD607	4.24: 1 (mg/L: mg/L)	7	TU/AI/MTI	7.42 mg/L	–	ADD^*^/ANT/ADD^*^	Zhou et al., 2018
	Zn (II) + Hg (II)	*E. coli* HB101pUCD607	1.41: 1 (mg/L: mg/L)	7	TU/AI/MTI	5.05 mg/L	–	ANT/ANT/ANT	Zhou et al., 2018
	Hg (II) + Cu (II)	*E. coli* HB101pUCD607	0.49: 1 (mg/L: mg/L)	7	TU/AI/MTI	4.88 mg/L	–	ADD^*^/ANT/ADD^*^	Zhou et al., 2018
	Hg (II) + Cu (II)	*E. coli* HB101pUCD607	0.16: 1 (mg/L: mg/L)	7	TU/AI/MTI	5.53 mg/L	–	ADD^*^/ANT/ADD^*^	Zhou et al., 2018
	Hg (II) + Cu (II)	*E. coli* HB101pUCD607	0.05: 1 (mg/L: mg/L)	7	TU/AI/MTI	0.72 mg/L	–	ANT/ANT/ANT	Zhou et al., 2018
Dibutyl phthalate and antibiotics	Chlorotetracycline hydrochloride + Dibutyl phthalate	*A. fischer*	EC_50_: EC_50_	–	TU	49.82 mg/L	0.9976	SYN	Wei et al., 2018
	Sulfamethazine + Dibutyl phthalate	*A. fischer*	EC_50_: EC_50_	–	TU	46.51 mg/L	0.9625	SYN	Wei et al., 2018
	Sulfamethazine + Dibutyl phthalate	*A. fischer*	EC_50_: EC_50_	–	TU	55.82 mg/L	0.9989	SYN	Wei et al., 2018
	Sulfadiazine + Dibutyl phthalate	*A. fischer*	EC_50_: EC_50_	–	TU	26.309 mg/L	0.9642	SYN	Wei et al., 2018
Antibiotics mixtures	Chlortetracycline hydrochloride + Sineptina	*P. phosphoreum*	EC_50_: EC_50_	7 ± 0.5	TU	0–2 TU	0.9610	SYN	Ren et al., 2011
	Chlortetracycline hydrochloride + Salinomycin	*P. phosphoreum*	EC_50_: EC_50_	7 ± 0.5	TU	0–2 TU	0.9613	ANT	Ren et al., 2011
	Chlortetracycline hydrochloride + Flavomycin	*P. phosphoreum*	EC_50_: EC_50_	7 ± 0.5	TU	0–2 TU	0.9554	ANT	Ren et al., 2011
	Sineptina + Salinomycin	*P. phosphoreum*	EC_50_: EC_50_	7 ± 0.5	TU	0–2 TU	0.9701	SYN	Ren et al., 2011
	Sineptina + Flavomycin	*P. phosphoreum*	EC_50_: EC_50_	7 ± 0.5	TU	0-2 TU	0.9794	SYN	Ren et al., 2011
	Salinomycin + Flavomycin	*P. phosphoreum*	EC_50_: EC_50_	7 ± 0.5	TU	0–2 TU	0.9872	ANT	Ren et al., 2011
Herbicidal mixtures	Fenuron + Simetryn	*P. phosphoreum*	EC_50_: EC_50_	–	TU	1.18 TU	–	ADD	Ge et al., 2014
	Fenuron + Atrazine	*P. phosphoreum*	EC_50_: EC_50_	–	TU	1.44 TU	–	ANT	Ge et al., 2014
	Fenuron + Prometon	*P. phosphoreum*	EC_50_: EC_50_	–	TU	2.28 TU	–	ANT	Ge et al., 2014
	Fenuron + Prometryn	*P. phosphoreum*	EC_50_: EC_50_	–	TU	2.52 TU	–	ANT	Ge et al., 2014
	Monuron + Simetryn	*P. phosphoreum*	EC_50_: EC_50_	–	TU	0.71 TU	–	SYN	Ge et al., 2014
	Monuron + Atrazine	*P. phosphoreum*	EC_50_: EC_50_	–	TU	2.84 TU	–	ANT	Ge et al., 2014
	Monuron + Prometon	*P. phosphoreum*	EC_50_: EC_50_	–	TU	1.09 TU	–	ADD	Ge et al., 2014
	Monuron + Prometryn	*P. phosphoreum*	EC_50_: EC_50_	–	TU	1.82 TU	–	ANT	Ge et al., 2014
	Monolinuron + Simetryn	*P. phosphoreum*	EC_50_: EC_50_	–	TU	0.90 TU	–	SYN	Ge et al., 2014
	Monolinuron + Atrazine	*P. phosphoreum*	EC_50_: EC_50_	–	TU	1.11 TU	–	ADD	Ge et al., 2014
	Monolinuron + Prometon	*P. phosphoreum*	EC_50_: EC_50_	–	TU	2.33 TU	–	ANT	Ge et al., 2014
	Monolinuron + Prometryn	*P. phosphoreum*	EC_50_: EC_50_	–	TU	1.44 TU	–	ANT	Ge et al., 2014
	Diuron + Simetryn	*P. phosphoreum*	EC_50_: EC_50_	–	TU	0.68 TU	–	SYN	Ge et al., 2014
	Diuron + Atrazine	*P. phosphoreum*	EC_50_: EC_50_	–	TU	1.22 TU	–	ANT	Ge et al., 2014
	Diuron + Prometon	*P. phosphoreum*	EC_50_: EC_50_	–	TU	2.59 TU	–	ANT	Ge et al., 2014
	Diuron + Prometryn	*P. phosphoreum*	EC_50_: EC_50_	–	TU	1.40 TU	–	ANT	Ge et al., 2014
Pesticides mixtures	Acifluorfen + Dimethoate + Prochloraz	*A. fischeri*	EC_50_: EC_50_: EC_50_	–	TU	0.66 TU		SYN	Cedergreen et al., 2012
	Acifluorfen + Diquat + Prochloraz	*A. fischeri*	EC_50_: EC_50_: EC_50_	–	TU	0.51 TU		SYN	Cedergreen et al., 2012
	Dimethoate + Prochloraz + Chlorfenvinphos	*A. fischeri*	EC_50_: EC_50_: EC_50_	–	TU	0.81 TU		SYN	Cedergreen et al., 2012
Heavy metal and other compounds	Cu + nitroaromatic compounds	*P. phosphoreum*	0.2 (Cu):1 (EC_50_: EC_50_) 0.5 (Cu):1 (EC_50_: EC_50_) 0.8 (Cu):1 (EC_50_: EC_50_)	–	TU/AI	At low Cu concentration, most binary mixtures show simple addition. Three binary mixtures are antagonistic. At medium Cu concentration, all effect types are existed. At high Cu concentration, the binary combined mixtures most showed antagonism, simple addition is only found for Cu combined with *o*-dinitrobenzene, *p-*nitrobromobenzene, *p*-nitrobenzoic acid and 2,4-dinitropheol			Su et al., 2012
Pesticides and MTBE	MTBE + Dichlofluanid/TBT/Linuron Diuron /Sea nine/Irgarol	*A. fischeri*	0.1 mg/L (MTBE): EC_50_ of individual pesticide	–	MTI	The combinations of MTBE and pure Diuron, Dichlofluanid, TBT, and Linuron are showed synergy. The addition of Sea nine and Irgarol did not increase the toxicity of the MTBE			Hernando et al., 2003

* *means the partly effect*.

*– means this item not mentioned in the article*.

*A1 method is the popular evaluation method which is mentioned as Equations (6) and (7), and A2 methods is showed as Equations (8) and (9)*.

All of the methods mentioned above are not considered the toxic mode of actions. The isobologram and response surface method are always addressed the binary toxicity of two-components mixtures. The toxic unit and corresponding methods except the statistical approach 2 (referred to TU_Diff_), assumed that no interaction among the components in the mixture. It is decisive to take advantages and disadvantages into consideration and choose the appropriate method for joint toxicity evaluation.

## Methods of Concentration Addition and Independence Action

### Concentration Addition Method

As reported by the EPA (EPA, 2000), the additivity is defined as “when the effect of the combination is estimated by the sum of the exposure levels or the effect of the individual chemicals,” In the context of the above definitions, the definitions about “effect” and “sum” draw forth two classical mathematical models. One is Concentration addition (CA, also termed Dose Addition or Loewe additivity), which assumes the constituents in a mixture has the same TMOA, differing in their efficacy. The joint effect of compound mixtures that have the same TMOA can be calculated using the CA model based on the concentration-response relationship of single substances (Loewe and Muischnek, 1926). The mathematical expression of CA by Equation (10):

(10) $$\begin{array}{l} \text{E}{\text{C}}_{\text{x,mix}}={\left(\overset{n}{\underset{i=1}{\sum }}\frac{p_{i}}{EC_{x,j}}\right)}^{-1} \end{array}$$

where EC_x,*mix*_ is the effective concentration of the mixture eliciting *x*% effect, EC_*x,i*_ denotes the concentration of the *i*th component when exists individually and elicits the same effect (*x*%) as the mixture, *p*_*i*_ is the molar concentration ratio of the *i*th component in the mixture.

**Figure 6:**
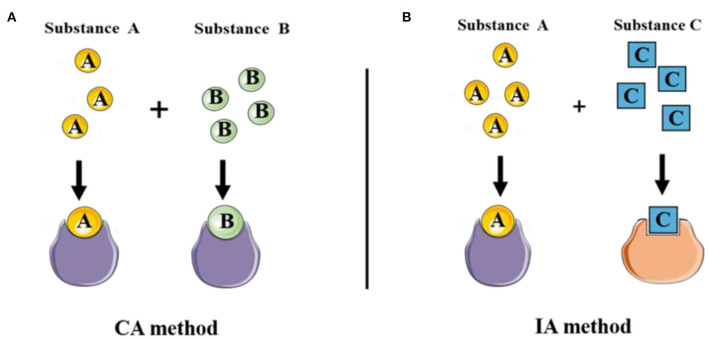
The diverse toxic mode of actions of classical joint prediction model of Concentration addition and Independent Action. **(A)** CA method was used to evaluate the joint toxicity of chemical substances with the same mechanism of action. **(B)** IA method was applied to evaluate the combined toxicity of multi-component with the different mechanisms of action.

CA implies that two (or more) components act on acceptors with common TMOA indiscriminately. That is to say, components can be substituted for each other in the mixtures (Figure 6A). The concept of CA hypothesized the combined effect may develop in the presence of multi-components. This hypothesis is significant because different categories of organisms in the ecosystem are exposed to a mixture of pollutants.

### Independent Action Method

In another case, the resistance of the organism to one component can not be replaced by another component in the mixture. That is to say, the response of two substances is statistically independent, the action mode of which belongs to different TMOAs (Figure 6B). The model IA model (or response addition) is another approach was hereby proposed to assess the mixture toxicity of components with dissimilar TMOAs, which means multi-component interaction with different target sites (Fraser, 1872; Bliss, 1939). IA model is commonly defined as:

(11) $$\begin{array}{l} E\left(c_{mix}\right)=1-\overset{n}{\underset{i=1}{\prod }}\left(1-E\left(c_{i}\right)\right) \end{array}$$

where *E*(*c*_*mix*_) is the overall effect caused by the total effect of the mixture, *c*_mix_ is the total concentration of a mixture; *E*(*c*_*i*_) is the value that denotes the single effect of the *i*th component corresponds to the concentration of *c*_*i*_in the mixture. The IA model is apt to predict the joint effect of mixtures with definite compositions.

### Which of the Two Models, CA or IA, Predict the Date More Precise?

From IA expression, we can conclude that toxicity assessment based on IA relies on the knowledge of the individual effect, which required from the concentration-response curves of all individual component. Moreover, *E*(*c*_*i*_) is lower than *E*(*c*_*mix*_) commonly (Junghans et al., 2006; Backhaus and Faust, 2012). That is, the limitation of IA may provoke the accuracy deviation of some predictions. Considering the simplicity ease to utilization, TU based on the CA model has been used in the context of unknown toxic effects of a single component.

There are partly researches were implemented to investigate the feasibility of CA and IA (Zhang et al., 2008; Liu et al., 2009; Zhou et al., 2010; Mo et al., 2014; Villa et al., 2014; Tong et al., 2015). It is not to say, CA (or IA) is only propitious to the similar (dissimilar) toxicology modes of action. The accuracy of CA even for the prediction of the toxicity of multi-component mixture composed with different TMOAs is investigated. Design uniform equivalent-effect concentration experimental method, the combined toxicity prediction of six organophosphorus pesticides (Fenitrothion, Malathion, Dicapthon, Chlormephos, Methyl parathion and Famphur) based on *V. qinghaiensis* sp.-Q67 can be precisely assessed by CA (Zhang et al., 2008). Others (Junghans et al., 2006; Zhou et al., 2010) also validated that CA may be a reasonable assumption for the toxicity of the multiple-component mixtures of pesticides regardless of the similarity or dissimilarity of their TMOA. Simultaneously, CA and IA are feasible for predicting the toxicological effect of some specific mixtures. Villa et al. tested the toxicity of complex mixtures of chemicals including narcotics, polar narcotics, herbicides, insecticides and fungicides toward *A. fisheri*. In this experiment, the set of chemicals was attributed to different groups on account of dissimilar toxicology modes of action. The finding demonstrated that CA and IA models are both feasible for predicting the toxicological effect of mixtures, and the difference between the two models was never higher than a factor of four (Villa et al., 2014). However, some researchers believe that the applicability of the two models remains a matter of debate in terms of accuracy in predicting the combined effects of mixtures. The potential risk management options should be studied more carefully (Carbajo et al., 2015; Godoy and Kummrow, 2017; Yang et al., 2017).

### Integrative Model of CA and IA

Some toxicants are composed of the components with similar or completely different toxic modes of action in the ecological environment. That is to say, some of which may have the same mode of action, while others may differ. Therefore, both the CA and the IA model may be not suitable for evaluating the combined toxicity of these mixtures. To work this out, the integrative models were developed to predict the toxicity of those complex mixtures (Ra et al., 2006; Qin et al., 2011; Kim et al., 2014; Mo et al., 2017). Among them, the TSP (two-stage prediction) model is one of the most commonly used approaches based on the integrative model concept (Mo et al., 2017). The basic principle of the TSP model is to apply the CA model and IA model that divided in stages to predict the joint toxicity of mixtures as following: In the first stage, the components in the mixture with similar TMOA are assigned into the same group, and joint toxicity of each group was calculated using CA model (Equation 10). In the second stage, the IA model is used to predict the mixture toxicity of all the groups with different TMOAs (Equation 11). In this way, the prediction of the combined toxicity of the mixture with similar and different TMOAs can be predicted.

Mo et al. selected six phenolic compounds (Methyl phenol, 2-Nitrophenol, 4-Nitrophenol, 2.4-Dichlorophenol, Phenol and 2-chlorophenol) and heavy metals (Cr, Cu, Ni, Cd, Ag, and Hg) with different TMOAs to compose the mixtures with *V. qinghaiensis* sp.-Q67, a series of mixtures were designed with equivalent-effect concentration and fixed concentration ration. By comparing the prediction error of CA, IA and TSP models, the TSP model exhibited better performance on predicting the overall effect of a mixture containing compounds with different TMOAs than CA and IA models (Mo et al., 2017). The predicted combined toxicity of mixtures of imidazolium and pyridinium ionic liquids in the ratios of their EC_50_, EC_10_, and NOEC on luciferase also showed that the TSP method preceded the CA and IA methods effectively (Ge et al., 2014).

The above methods are both attributed to traditional experimental methods, with the aid of the individual concentration-effect relationship so as the final judgment of the multi-component mixture can be confirmed. Moreover, experimental toxicology studies usually used experimental animals, tissues, bacteria, cell. It is no doubt these requirements have improved the complexity of the operations. Hence, researchers are devoted to developing alternative methods to conquer these limitations of traditional experimental methods. QSAR (Roy et al., 2015a,b), Read-across (RA) (Jeliazkova et al., 2010), Molecular docking (Yao et al., 2013) and Expert systems (Roy and Kar, 2016), are deemed faster, cheaper and can manifest more information (Mwense et al., 2004). In these developing alternative methods, the QSAR model is one of the most widely used methods *in silico*.

### QSAR Assisted Toxicity Prediction

QSAR, as a calculated method has been widely applied in toxicology. QSAR is an acronym for Quantitative Structure-Activity Relationship. The concept was delivered in 1872, and the modern age of QSAR analysis originated from the works of Hansch et al. (1962). By mathematical function, QSAR is a statistical approach which express a relationship between the magnitude of biological effect (BA) and changes in a molecular structure, as Equation (12) showed:

(12) $$\begin{array}{l} \text{B}{\text{A}}_{i}=f\left(X_{i}\right) \end{array}$$

where *i* denotes a specific chemical of a series, this series may be of homogeneous, or of heterogeneous substances.

In the study of constructing a QSAR model, it is decisive to acquire and screen molecular structure descriptors (also called indicators). At present, more than 3,000 molecular descriptors have been defined and applied to QSAR models to predict group biotoxicity. Choose distinct indicators when adopting the QSAR model that will affect the validity of the predicted results, therefore the preparation of the indicators is the key to the construction (Khan et al., 2020). Among abundant kinds of indicators, the partition coefficient of a chemical between *n*-octanol and water is supposed to be the most effective indicator (Tichy et al., 2008). However, it is difficult to use *K*_ow_ to determine the combined toxicity of mixtures, since the available date was obtained by UV spectrophotometry or HPLC is only suitable for determining the *K*_ow_ of single chemicals. Furthermore, Verhaar extended the C_18_-Empore^TM^ disks/water partition coefficient (*K*_MD_) to predict the bioconcentration of mixtures, which was found to have a close relationship with log*K*_ow_ (Verhaar et al., 1995). The C_18_-Empore^TM^ disks/water partition coefficient (*K*_MD_) can be calculated from Equation (13), the unmeasurable problem was successfully solved.

(13) KMD=WV×{∑i=1nQwater,i01+(WVKSDi)/ (∑i=1nQwater00−∑i=1nQwater,i01+(WVKSDi))}

where *W* is the volume of solution, *V* is the volume of hydrophobic phase, is the initial amount of chemical *i* in water, *n* is the total number of individual chemicals in the mixture, and *K*_SDi_ is the partition coefficient of individual chemical *i*. The value of *W/V* was suggested to be 6.8 × 10^5^.

Not limited to the partition coefficient of chemical between *n*-octanol and water, studies concerned other kind indicators to employ QSAR to assess ecotoxicity evaluations of diverse pollutants toward familiar luminescent bacteria, *A. fisheri, P. phosphoreum* and *V. qinghaiensis* sp*.-*Q67, which are categorically reviewed as shown in Table 4.

**Table 4:** Characterizations of various types of QSAR models for prediction of joint toxicity.

**Indicators**	**Components**	**Models**	**Test organism**	**References**
log*K*_ow_	Narcotics	lg1/*C*_narc_(mmol/L) = 0.94log*K*_ow_ − 2.61	*A. fischeri*	Vighi et al., 2009
		*n* = 23, *R*^2^ = 0.92		
	Polar narcotics compounds	lg1/*C*_polar_(mmol/L) = 0.502log*K*_ow_ + 0.294	*A. fischeri*	Vighi et al., 2009
		*n* = 25, *R*^2^ = 0.82		
*K*_MD_	Halogenated benzenes	lg*EC*_50_ = 0.928log*K*_MD_ + 0.224	*P. phosphoreum*	Lin et al., 2002
		*n* = 74, *R*^2^ = 0.973, *SE* = 0.113, *F* = 287.785, *P* < 0.001		
*E*_bind_	Sulfonamides (SA) –SA potentiators (SAP)	$-\mathrm{lg}EC_{\text{50}}=3.752-0.030\times \left(C_{\text{SA}}/\sum C\right)\times E_{\text{bind}}^{\text{SA}}-0.045\times \left(C_{\text{SAP}}/\sum C\right)\times E_{\text{bind}}^{\text{SAP}}$[[Mathtype-mtef1-eqn-40.mtf]]	*A. fischeri*	Wang et al., 2018
		*n* = 18, *R*^2^ = 0.699, RMSE = 0.161, *F* = 17.432, *P* = 0.000, Q^2^loo = 0.600, RMSEloo = 0.174, $Q_{F_{1}}^{2}=0.770$, RMSEP = 0.067		
*E*_bind_	Sulfonamides (SA) –tetracyclines (TC)	$-\mathrm{lg}EC_{\text{50}}=5.419+0.042\times \left(C_{\text{SA}}/\sum C\right)\times E_{\text{bind}}^{\text{SA}}+0.042\times \left(C_{\text{TC}}/\sum C\right)\times E_{\text{bind}}^{\text{TC}}$	*A. fischeri*	Wang et al., 2018
		*n* = 15, *R*^2^ = 0.813, RMSE = 0.068, *F* = 26.138, *P* = 0.000, Q^2^loo = 0.714, RMSEloo = 0.075, $Q_{F_{1}}^{2}$= 0.601, RMSEP = 0.039		
*E*_bind_	SA potentiators (SAP) –tetracyclines (TC)	$-\mathrm{lg}EC_{\text{50}}=4.852-0.031\times \left(C_{\text{SAP}}/\sum C\right)\times E_{\text{bind}}^{\text{SAP}}+0.024\times \left(C_{\text{TC}}/\sum C\right)\times E_{\text{bind}}^{\text{TC}}$	*A. fischeri*	Wang et al., 2018
		*n* = 8, *R*^2^ = 0.829, RMSE = 0.092, *F* = 12.109, *P* = 0.012, Q^2^loo = 0.603, RMSEloo = 0.111,		
		$Q_{F_{1}}^{2}$= 0.760, RMSEP = 0.069		
*E*_HOMO_, *E*_LUMO_	2,4 Dinitrotoluene –aromatic compounds	−lg(1/*EC*_50_) = 3.672−0.882*E*_LUMO_	*A. fischeri*	Yuan et al., 2002
		*n* = 8, *r* = 0.925, *s* = 0.406		
*E*_bind_*, pK*_a_	Sulfonamide antibiotics mixtures	$-\mathrm{lg}EC_{\text{50}}=2.318+0.118\times pK\text{a}+0.027\times \left(C_{\text{i}}^{\text{SA}}/\sum C\right)\times E_{\text{bind}}^{\text{SA}}-0.001\times \left(C_{\text{i}}^{\text{TMP}}/\sum C_{i}\right)\times E_{\text{bind}}^{\text{TMP}}$	*P. phosphoreum*	Zou et al., 2012
		*n* = 7, *R*^2^ = 0.954, *SE* = 0.028, *F* = 20.659, *P* = 0.017		
*E*_HOMO_*, E*_LUMO_	(0.2 × EC_50_) Cd –chlorinated anilines	lg*EC*_50_ = −28.212(*E*_HOMO_ − *E*_LUMO_)−4.187	*P. phosphoreum*	Jin et al., 2014
		*n* = 9, *R*^2^ = 0.832, *SE* = 0.0603, *F* = 34.769, *P* = 0.001, $Q_{LOO}^{2}=0.7769$, RMS_CV_ = 0.064		
*E*_HOMO_*, E*_LUMO_	(0.5 × EC_50_) Cd –chlorinated anilines	lg*EC*_50_ = −42.770(*E*_HOMO_ − *E*_LUMO_)−7.243	*P. phosphoreum*	Jin et al., 2014
		*n* = 9, *R*^2^ = 0.837, *SE* = 0.0900, *F* = 35.87, *P* = 0.001, $Q_{LOO}^{2}=0.7774$, RMS_CV_ = 0.094		
*E*_HOMO_*, E*_LUMO_	(0.8 × EC_50_) Cd –chlorinated anilines	lg*EC*_50_ = −55.638(*E*_HOMO_ − *E*_LUMO_)−9.791	*P. phosphoreum*	Jin et al., 2014
		*n* = 9, *R*^2^ = 0.903, *SE* = 0.0869, *F* = 65.18, *P* = 0.000, $Q_{LOO}^{2}$ = 0.8204, RMS_CV_ = 0.107		
*CSEV, S*	(0.2 × EC_50_) Cu –nitroaromatic compounds	lg1/*EC*_50_ = −1.459+0.043*CSEV*+0.684*S*	*P. phosphoreum*	Su et al., 2012
		*n* = 11, *R*^2^ = 0.828, *SE* = 0.251, *F* = 19.2, *P* = 0.001		
*CAA*	(0.5 × EC_50_) Cu –nitroaromatic compounds	lg1/*EC*_50_ = −8.039+0.043*CAA*	*P. phosphoreum*	Su et al., 2012
		*n* = 11, *R*^2^ = 0.727, *SE* = 0.411, *F* = 25.0, *P* = 0.001		
*CAA*	(0.8 × EC_50_) Cu –nitroaromatic compounds	lg1/*EC*_50_ = −8.754+0.046*CAA*	*P. phosphoreum*	Su et al., 2012
		*n* = 11, *R*^2^ = 0.732, *SE* = 0.432, *F* = 24.6, *P* = 0.001		
*VE2_B(p)* TIC3	(0.2 × EC_50_) Zn–nitro-substituted benzenes	lg1/*EC*_50_ = 20.540−53.948*VE*2_*B*(*p*)−0.033*TIC*3	*P. phosphoreum*	Zhang et al., 2019a
		*n* = 11, *R*^2^ = 0.933, *SE* = 0.163, *F* = 55.554, *P* < 0.001		
*Eig06_AEA(dm)*	(0.5 × EC_50_) Zn–nitro-substituted benzenes	lg1/*EC*_50_ = 3.760+1.100*Eig*06_*AEA*(*dm*)	*P. phosphoreum*	Zhang et al., 2019a
		*n* = 11, *R*^2^ = 0.856, *SE* = 0.232, *F* = 53.318, *P* < 0.001		
*Eig06_AEA(dm)*	(0.8 × EC_50_) Zn –nitro-substituted benzenes	lg1/*EC*_50_ = 3.908+1.511*Eig*06_*AEA*(*dm*)	*P. phosphoreum*	(Zhang et al., 2019a
		*n* = 11, *R*^2^ = 0.937, *SE* = 0.201, *F* = 13.351, *P* < 0.001		
$\rho_{\max\left(\text{H}\right)}^{+}$	Benzene—its derivatives	$\mathrm{lg}1/EC_{50}=13.119\left(\pm 3.202\right)\bar{\rho_{\max\left(\text{H}\right)}^{+}}-22.744\left(\pm 3.065\right)\bar{1/APSA}+10.25\left(\pm 0.91\right)$	*A. fischeri*	Chang et al., 2016
		*n* = 40, *R*^2^ = 0.827, $R_{cv}^{2}=0.826$, *SD* = 0.269, $R_{adj}^{2}=0.849$, *F* = 110.935, *P* < 0.0001.		
σ_*p*_, C^*^	Aldehyde –cyanogenic toxicant	$M=0.367-0.811\sigma_{\text{P}}-6.704C^{*}$	*P. phosphoreum*	Lin et al., 2005
		*R*^2^ = 0.868, *SE* = 0.232, *F* = 121.769, *P* = 0.000, M is the sum of toxic units		
*O*_aldehyd_, *C*_cyanogenic_	Aldehyde –cyanogenic toxicant	TU = 1.00 ± 0.20 when *O*_aldehyde_- *C*_cyanogenic_ >-0.125	*P. phosphoreum*	Tian et al., 2012a
		TU = −27.6 × *O*_aldehyde_ − 5.22 × *C*_cyanogenic_ − 6.97 when *O*_aldehyde_- *C*_cyanogenic_ ≤ -0.125		
		*n* = 40, *r* = 0.887, *SE* = 0.195, *F* = 140, *p* < 0.001, $Q_{LOO}^{2}$ = 0.748		
log *K*_mbw_, log *K*_mcw_	Nonpolar/polar—narcotic—chemical mixtures	$\log1/EC_{50}=1.086+0.830\log K_{\text{mow}}+0.527A^{\text{MH}}$, $A^{\text{MH}}=\log K_{\text{mbw}}-\log K_{\text{mcw}}$	*P. phosphoreum*	Lin et al., 2003
		*n* = 84, *R*^2^ = 0.948, *SE* = 0.166, *F* = 745.201, *p* = 0.000		
*RDF035m HATSs H-047*	Antibiotic and pesticides	*pEC*_50,*mix*_ = (2.2780 ± 0.2173)−(0.0546 ± 0.0050) × (*RDF*035*m*)_*mix*_ −(0.0158 ± 0.0024) × (*HATSs*)_*mix*_ − (0.3124 ± 0.0214) × (*H*−047)_*mix*_	*A. fischeri*	Qin et al., 2018
		*n* = 31, *R*^2^ = 0.9366, RMSE = 0.1345, *F* = 132.89, $Q_{LOO}^{2}$ = 0.9087, $R_{adj}^{2}$= 0.9087		
*Mor13s, L/Bw, Eig08_EA(ed) Eig09_EA(ed)*	Aromatic brominated –chlorinated disinfection byproducts	*pEC*_50,*mix*_ = 3.7370(±0.0324)−0.1710(±0.0135)*M*or13*s*−0.0421(±0.0135)*L*/*Bw* +0.0866(±0.0093)*Eig*08_*EA*(*ed*)−0.0085(±0.0017)*Eig*09_*EA*(*ed*)	*V. qinghaiensis sp.-*Q67	Chen et al., 2019
		*n* = 31, $Q_{F1}^{2}$ = 0.7804, $Q_{F2}^{2}$ = 0.7782, $Q_{F3}^{2}$ = 0.8057, *CCC* = 0.8778, $Q_{LOO}^{2}$ = 0.7700		
*R_7__TPSA, R_2__pK_*a*_*,	Natural deep eutectic solvents	log*EC*_50_ = −5.596*R*7_*TPSA*+0.145*R*2_*pK*_*a*_	*A. fischeri*	Giner et al., 2020
		*R*^2^ = 0.858, *SE* = 0.166, *F* = 103, *P* < 0.0001		

Researches about QSAR are still of significant interest in the development of innovative models in environmental toxicity prediction. The emergence of the QSAR models fills the gap in predicting the combined toxicity of organic compounds and heavy metals since the information in this field is still scarce. Jin et al. developed three QSARs to determine the individual EC_50_ of Cd and nine chlorinated anilines (*o*-chloroaniline, *m*-chloroaniline, *p*-chloroaniline, 2,3-dichloroaniline, 2,4-dichloroaniline, 2,5-dichloroaniline, 2,6-dichloroaniline, 3,4-dichloroaniline, and 2,4,5-trichloroaniline) with *P. phosphoreum*, setting three different levels of Cd concentrations (low, medium and high levels) to mix with chlorinated anilines and the results showed that the number of chlorinated anilines manifesting synergy with Cd is decreasing as the concentration of Cd increases. The robustness of the models was confirmed by comparing the experimental and predicted values, and all the relative error values remain within 16% (Jin et al., 2014). Likewise, the joint toxicities of Cu (low, medium and high levels) with 11 nitroaromatic compounds (nitrobenzene, *o*-dinitrobenzene, *m*-nitrobromobenzene, *p*-nitrobromobenzene, *o*-nitroaniline, *p*-nitroaniline, *p*-nitrobenzoic acid, *o*-nitrophenol, *m*-nitrophenol, *p*-nitrophenol and 2,4-dinitrophenol) were studied by *P. phosphoreum* with developed QSAR analysis, there is a good agreement between the predicted values and experimental with *R*^2^ = 0.764, *P* = 0.000, (Su et al., 2012). By drawing on the study of Su's, Zhang's, Su, Zhang, Li, Qin and Zhang (2019a) work on the acute toxic effect of mixtures between metal Zn and above-mentioned 11 nitroaromatic compounds established robust QSAR models to predict the joint interaction when combined with Zn at low, medium, and high concentrations (Zhang et al., 2019a). Not limited to heavy metals and organics, the joint toxicity of mixed organics can also be predicted by QSAR models. Qin et al. developed a generalized QSAR model for predicting the additive and non-additive toxicities of multi-component mixtures, the experiment tested the joint toxicity of 45 multi-component mixtures composed of two antibiotics (Tetracycline hydrochloric and Chloramphenicol) and four pesticides (Metribuzine, Trichlorfon, dichlorvos, Linuron) to *A. fisheri*. Compared with classical CA and IA models, the result demonstrated that the QSAR model exhibited high predictive capability for predicting joint toxicity (Qin et al., 2018).

### The Potential Mechanisms of Joint Toxicity

Though more and more efforts have been devoted to investigating mechanisms with mixture toxicity, joint toxicity mechanisms, especially for specific pollutants, are still too complex to figure out. In recent years, there has been growing evidence regarding the experimental factors including mixture ratio setting and complexity of toxic compounds play a vital role in shaping the joint effect, which in turn impacts the determination of toxicological mechanism. At the same time, considering the environmental factors on the joint effect study, the ecological environmental media should be researched more adequately. Because of the above-mentioned reasons, the studies concentrated on the mechanisms of joint toxicity toward luminescent bacteria are merely few. Analogous to other test organisms, impair mechanisms of the luminescent bacteria are speculated in this section.

### Trojan-Horse Effect and Reverse Trojan-Horse Effect

**Figure 7:**
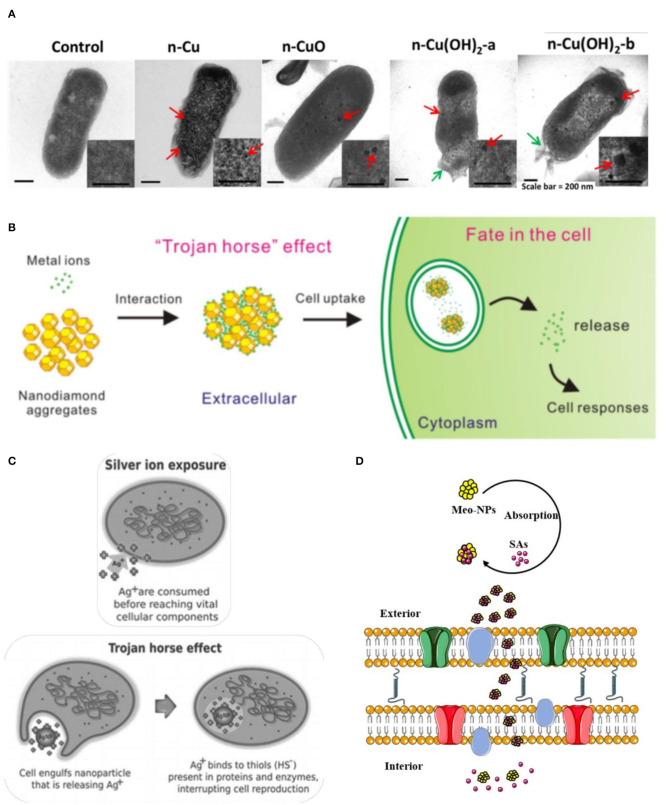
The potential mechanism of Trojan-horse type. **(A)** High—resolution transmission electron microscopy (TEM) micrographs of *E. coli* cells treated with nano Cu to CuO and Cu(OH)_2._ Red arrows indicated Cu particles; green arrows indicate membrane damage (Kaweeteerawat et al., 2015). **(B)** The scheme of absorption of metal ions on Nanodiamond (NDs) led to cellular toxicity (Zhu et al., 2015). **(C)** Comparison of silver ion and AgNP exposure, the toxicity of AgNP are more toxic than those of silver ions by themselves because the ions would be largely cvnonsumed in the process of penetration, which chimed in with the Trojan horse effect (Quadros and Marr, 2012). **(D)** Meo-NPs act like Trojan-horse to deliver SAs into the inner cell membrane (Wang et al., 2016). Adapted and modified with permission from Kaweeteerawat et al. (2015), Zhu et al. (2015), Quadros and Marr (2012), and Wang et al. (2016).

The cellular membrane acts as a significant site for interactions of chemicals. A wealth of reports showed that physiological activities in the inner of an organism are related to the cellular membranes. The functions of the cell membrane are considered in many aspects, such as transmembrane transport of small molecules, energy exchange, cell recognition, cell-mediated immunity, nerve conduction and metabolism regulation and so on (Oxender and Fox, 1978; Alberts et al., 1989). The impact of NPs on the membrane or wall integrity of algal cells (Angel et al., 2015; Sendra et al., 2017; Sousa et al., 2019) and bacteria cells (Kaweeteerawat et al., 2015; Martín-de-Lucía et al., 2017; Pulido-Reyes et al., 2017) (Figure 7A), has been evaluated extensively. Indications of damaged cell membrane make it interesting to study the potential biotoxicity effect of multi-component mixtures. A principal effect hypothesis concerning the cellular membrane damage which is described as the “Trojan-horse effect” has garnered considerable attention (Limbach et al., 2007). This hypothesis was first proposed and adopted gradually in nanomaterial. Owing to the large surface area, the ability of nanomaterials to act as carriers for a wide range of heavy metals and organic pollutants (Liu et al., 2018). CNTs and C_60_ are confirmed to carry heavy metals and dichlorodiphenyldichloroethylene (*p, p*'-DDE) entry into the inner cell via damaging cell membranes, respectively. Except for membrane disruptions, the entry process can impair other intracellular structures (receptors, ion channels, transporters, glycoproteins) (De La Torre-Roche et al., 2012; Wang et al., 2015a). The interaction toxicity of the Pb-nanotube also showed a synergistic effect, increasing over five times compared with the toxicity of single Pb (Martinez et al., 2013). Carbon adsorption of pollutants by nanomaterials was common in other organisms, like Japanese medaka (Su et al., 2013), *Daphnia magna* (Simon et al., 2015), *Cyprinus carpio* (Zhang et al., 2007). Based on the Trojan-horse effect, the schematic illustration of adsorption of metals ions on nanodiamonds (NDs) and the AgNP (Silver Nanoparticles) exposure, were shown in the Figures 7B,C (Quadros and Marr, 2012; Zhu et al., 2015). That mechanism also applied to luminescent bacteria, which elucidated that the synergistic effect of sulfonamides (SAs) with metal oxide nanoparticles (Meo-NPs) toward *A. fischer* (Figure 7D; Wang et al., 2016). As absorbent and carrier, metal oxide nanoparticles facilitated the entry of SAs into the inner of membrane completely and finally upon reaching the target site and release the encapsulated SAs molecules by making use of local physiological stimuli present here.

At the opposite extreme from the “Trojan-horse effect,” the term “Reverse Trojan-horse effect” is a relatively new name for referring to describing these phenomenons that antagonism is the predominant effect of multi-component mixtures (Barranger et al., 2019). Using *A.fisheri*, Sanchiss' study of the combined ecotoxicity of fullerene-soot and co-contaminants as malathion, diuron, triclosan, and nonylphenol indicated the joint effect of antagonism in all of the cases (Sanchis et al., 2016). In their analysis of this result, Sanchis et al. mentioned the special situation of aromatic rings as a tentative substance of allowing the production of micelles and changing the state of aggregation of fullerene aggregates. Therefore, the simple surface sorption process cannot clarify the antagonism between the fullerene-soot and co-contaminants. Interestingly antagonism appeared at low effect levels in wastewater–nanoparticle mixtures using recombinant bioluminescent cyanobacterium *Anabaena* sp. PCC 7120 strain CPB4337 (Martín-de-Lucía et al., 2017). Furthermore, the other four categories were added by Naasz et al. to discriminate different categories to recommend the joint toxicity upon mixture exposure, the additional mechanisms that are Surface enrichment, Retention, Inertism and Coalism (Naasz et al., 2018).

### Competition for the Active Site

In the plasma membrane, some transmembrane proteins serve as receptors to detect and transduce chemical signals in the cell environment. According to the receptor theory, toxic targets are mainly receptors on the cell surface or in the nucleus and cytoplasm (Wu et al., 2016). The receptor theory represented that one pollutant with better absorption may replace those with poor adsorption when pollutants with similar chemical properties compete on the cell surface that will cause the specific biological accumulation and biotoxicity (Elliott et al., 1986; Nirmalakhandan et al., 1994; Posthuma et al., 1997). A study on the mixture toxicity of heavy metals demonstrated that the majority of sites were already immobilized by ions to the extent that new chemicals hardly have a chance to bind, such a strategy may fluctuate the joint effect along with competition for toxic sites among coexisting cations (Zeng et al., 2015).

### Other Factors Affect Toxicity Prediction

**Figure 8:**
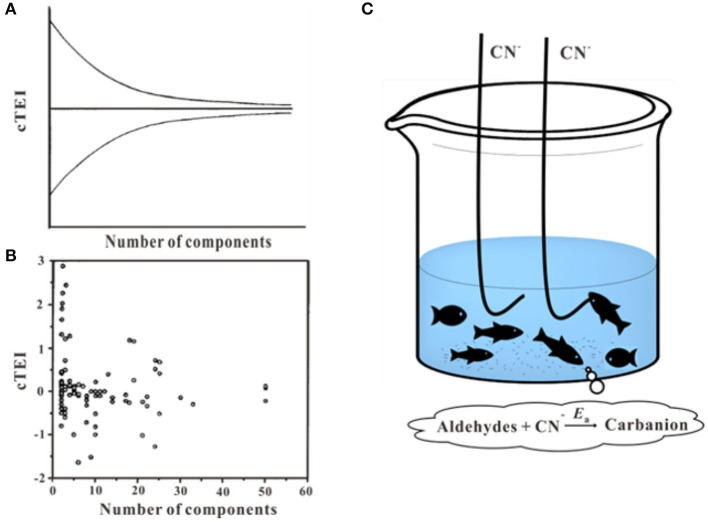
Funnel hypothesis and fishing hypothesis. **(A,B)** Toxicity enhancement index values (cTEI) plotted against the number of components in the mixtures (Warne and Hawker, 1995). **(C)** Schematic of the fishing hypothesis (Li et al., 2014). Adapted and modified with permission from Warne and Hawker (1995) and Li et al. (2014).

It has been mentioned, the joint effect of chemical mixtures varies due to its type of compounds and toxic ratios (at their equitoxic ratios or non-equitoxic ratios). The multi-chemical mixture may show dissimilar toxicity response with a different ratio of the same chemicals (Kar and Leszczynski, 2019). For narcotic toxicants, funnel hypothesis was put forward to interpret the fact that as the number of components in a mixture increase, the range of deviation from toxic additivity decreases (Rayburn et al., 1991; Warne and Hawker, 1995) (Figures 8A,B). With the number of components increases, the joint effect tends to show additivity effect and toxicity reduction can be regarded as a result of dissolution and dilution (Altenburger et al., 2000; Backhaus et al., 2000; Faust et al., 2001). The equitoxic mixtures of the reactive chemicals also have the same tendency, mixtures effect is close to additive as the increased number of the components (Tian et al., 2012b). It is necessary to note that there are inconsistent with funnel hypothesis as some ternary mixtures effect showed more synergistic or antagonistic than binary mixtures effect (Cedergreen et al., 2012; Chen et al., 2014, 2015; Wang et al., 2015b,c). Besides, the fishing hypothesis was proposed to explain the variation rules of the joint effects of cyanogenic toxicants and aldehydes (Figure 8C; Li et al., 2014). The hypothesis elucidated why the interactions based on the equitoxic ratios of the different chemicals were stronger than the effects at non-equitoxic ratios. In this hypothesis, the CN^−^ acted as fishhooks and aldehydes acted as fish. A hook only caught one fish when fishing. There is a competition among different types of fish for one hook during the process of fishing, while no competitions among different types of fish. However, there is no uniform explanation for the prediction of different types of interactions in the multi-component mixtures, which remain a rough challenge for ecotoxicology.

## Conclusion and Future Perspectives

Compared with other biological assay methods, luminescent bacteria toxicity assay has the advantages of simple operation, short test time and high repeatability. The results of numerous researches have illustrated the joint effect of multi-component mixtures and described the various types of models involved in joint toxicity prediction based on bacteria toxicity assay. Other studies also attempt to evaluate the toxicity of specific toxins by combining luminescent bacteria with other means such as dual detections by a combination of luminescent bacteria and probe. However, the effect of major factors such as the number of mixed components, the dominating components, and the toxic ratio of individual toxicants, on the joint effect of multi-component mixtures should be considered necessary. To address variable co-exposure scenarios, further research is required to decipher more related indicators to construct a fitting model for the assessment of multi-component mixtures. It would contribute to promoting thus further use of effect-based prediction of ecological environmental quality. Furthermore, the current luminescent bacteria toxicity assay is mostly used for acute toxicity testing, the determination of chronic toxicity exposure based on luminescent bacteria should be refined within the larger regulatory network. The differences between chronic and acute toxicity require further elucidation.

## Author Contributions

All authors wrote the manuscript jointly. DW, SW, and LB contributed to reviewing the literature and the write up of the manuscript. MN, SL, and WY contributed to the editing of the manuscript.

## Conflict of Interest

The authors declare that the research was conducted in the absence of any commercial or financial relationships that could be construed as a potential conflict of interest.
